# Stop-codon read-through arises largely from molecular errors and is generally nonadaptive

**DOI:** 10.1371/journal.pgen.1008141

**Published:** 2019-05-23

**Authors:** Chuan Li, Jianzhi Zhang

**Affiliations:** Department of Ecology and Evolutionary Biology, University of Michigan, Ann Arbor, MI, United States of America; Université Claude Bernard - Lyon 1, FRANCE

## Abstract

Stop-codon read-through refers to the phenomenon that a ribosome goes past the stop codon and continues translating into the otherwise untranslated region (UTR) of a transcript. Recent ribosome-profiling experiments in eukaryotes uncovered widespread stop-codon read-through that also varies among tissues, prompting the adaptive hypothesis that stop-codon read-through is an important, regulated mechanism for generating proteome diversity. Here we propose and test a competing hypothesis that stop-codon read-through arises mostly from molecular errors and is largely nonadaptive. The error hypothesis makes distinct predictions about the probability of read-through, frequency of sequence motifs for read-through, and conservation of the read-through region, each of which is supported by genome-scale data from yeasts and fruit flies. Thus, except for the few cases with demonstrated functions, stop-codon read-through is generally nonadaptive. This finding, along with other molecular errors recently quantified, reveals a much less precise or orderly cellular life than is commonly thought.

## Author summary

The stop codon gives the translating ribosome the signal for the termination of peptide synthesis, but occasionally the ribosome goes past the stop codon and continues translating into the otherwise untranslated region of a transcript. Stop-codon read-through generates an elongated peptide, which could be beneficial under certain circumstances. Although stop-codon read-through was thought to be rare, recent ribosome-profiling experiments in eukaryotes discovered hundreds of genes that undergo stop-codon read-through at a detectable rate. It is unclear whether most of these observed read-through events have biological functions or reflect cellular errors. The error hypothesis makes a set of distinct predictions about the probability of read-through, frequency of sequence motifs for read-through, and conservation of the read-through region. Our analysis of genome-scale data from yeasts and fruit flies verifies each of these predictions, suggesting that most stop-codon read-through events are nonadaptive cellular errors. These and related findings of various molecular errors in transcription and posttranscriptional modification paint a much less precise or orderly cellular life than is commonly portrayed.

## Introduction

In the standard genetic code, three (TAA, TAG, and TGA) of the 64 codons are stop codons, which, unlike sense codons, do not have corresponding tRNAs. These stop codons are instead recognized by release factors, causing the translating ribosome to terminate peptide synthesis and be released from the transcript. Occasionally, however, the ribosome may incorporate a standard or specialized amino acid at the stop codon and translate into the normally untranslated region (UTR) of the transcript until encountering the next stop codon [1]. This phenomenon is known as stop-codon read-through.

Because stop-codon read-through extends the C-terminus of a protein, it can alter the protein function, which could be beneficial in some cases. For instance, stop-codon read-through is a common strategy of viruses to encode proteins with an extended C-terminus [2]. A well-described example is the *gag*/*pol* translational read-through in retroviruses, where a ~5% probability of read-through of the *gag* UAG stop codon is required to form the *gag*-*pol* polyprotein necessary for virion assembly [3]. Stop-codon read-through is also known in eukaryotes [4] and can influence protein localization by adding a signal peptide [5–7].

Stop-codon read-through had been thought to be rare until recent ribosome-profiling experiments that showed otherwise [8, 9]. These experiments sequence all mRNA segments protected by ribosomes in a transcriptome at a given moment, revealing each mRNA segment that is being translated as well as the relative number of ribosomes that are translating the segment [10]. For instance, in fruit flies, hundreds of genes have 3’ UTRs protected by ribosomes, revealing widespread stop-codon read-through [9]. In addition, the set of genes subject to stop-codon read-through varies among fruit fly tissues/cell types, and read-through regions exhibit slightly but significantly higher sequence conservation than their downstream untranslated regions [9]. These observations led to the assertion that stop-codon read-through is an important, regulated mechanism for generating proteome diversity [9], a view shared by other researchers of stop-codon read-through [11–13]. Furthermore, certain stresses induce stop-codon read-through, creating altered protein functions that could be advantageous in stressful environments [14, 15]. Hence, stop-codon read-through is thought to have been selectively maintained in evolution as a mechanism promoting evolvability [16].

Notwithstanding, the possibility exists that stop-codon read-through, like many other processes that can generate transcriptome and proteome diversities (e.g., RNA editing and alternative polyadenylation), primarily reflects molecular errors and are nonadaptive [17–22]. In this work, we test the error hypothesis using ribosome-profiling data from the yeast *Saccharomyces cerevisiae* and the fruit fly *Drosophila melanogaster* along with other genomic data. We show that the error hypothesis makes multiple distinct predictions that are all supported by the data analyzed.

## Results

### Read-through rates decrease with gene expression levels

Stop-codon read-through is expected to be mostly deleterious if it largely originates from molecular errors. The potential deleterious effect could arise from (i) a reduction in the fraction of protein molecules with normal functions, (ii) a waste of cellular resource and energy in protein synthesis, and (iii) a gain of protein molecules with toxicity. Let the rate of stop-codon read-through be the probability that a ribosome reads through the stop codon in a run of translation. Given this rate, the deleterious effect from (ii) and (iii) rises with the number of protein molecules synthesized. Hence, natural selection against stop-codon read-through in a gene intensifies with the mRNA concentration of the gene. Consequently, the above defined rate of stop-codon read-through should decrease with the gene expression level. By contrast, the adaptive hypothesis of stop-codon read-through does not predict this negative correlation *a priori*, because, under this hypothesis, the rate of stop-codon read-through in a gene should depend on the specific function of the elongated protein.

**Fig 1:**
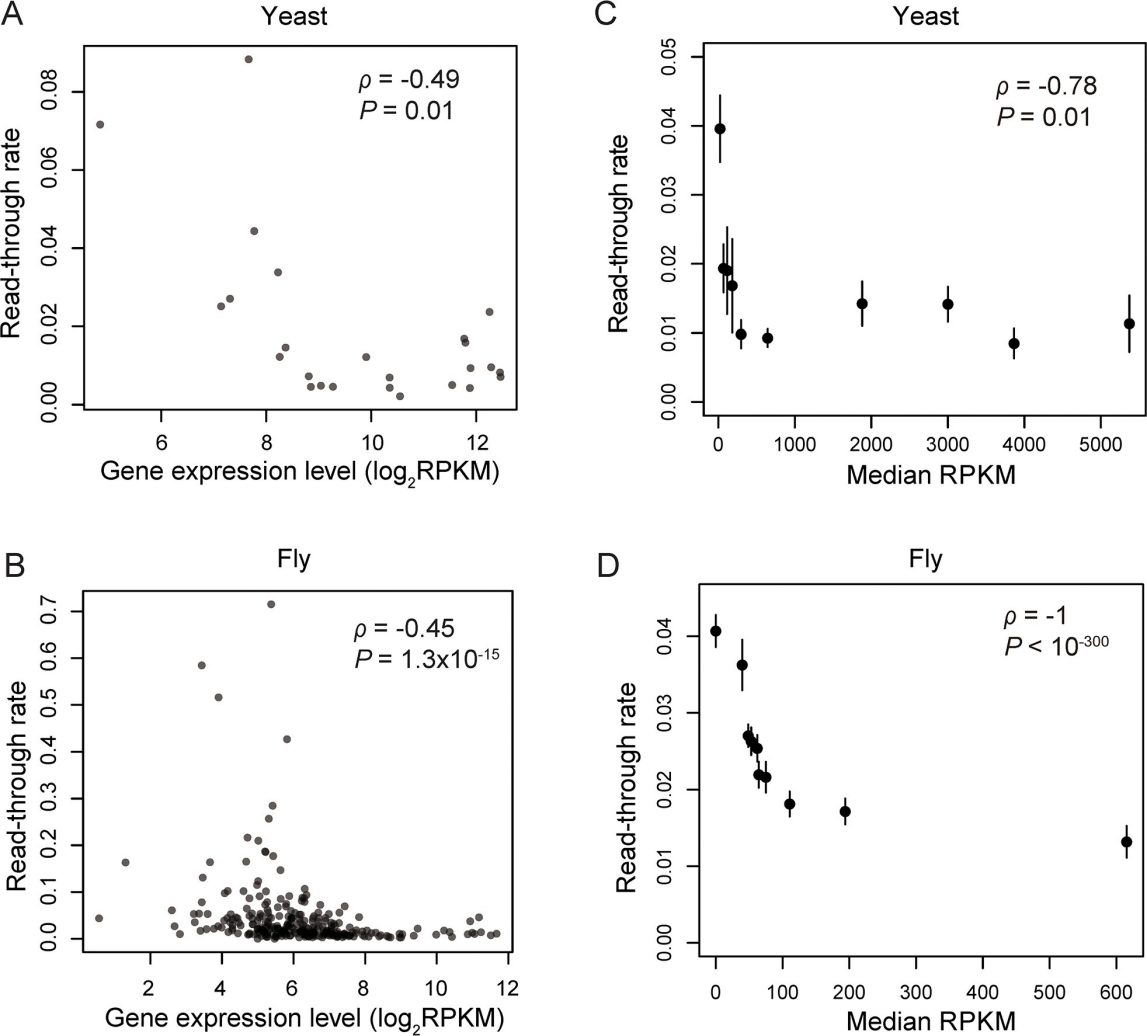
Stop-codon read-through rates decrease with gene expression levels. The read-through rate is estimated by the ribosome profiling RPKM of region 1 relative to that of the coding region. Gene expression level is measured by log_2_(RPKM) of the coding region based on mRNA sequencing. (**A-B**) The read-through rate of a gene declines with its expression level among 25 genes in yeast (**A**) and 283 genes in fruit fly (**B**) reported to undergo stop-codon read-through in [9]. Each dot is a gene. (**C-D**) The overall read-through rate of a group of yeast (**C**) or fruit fly (**D**) genes of similar expression levels declines with the median expression level of the group of genes. In each species, genes are divided into 10 bins according to their expression levels such that the total expression level (measured by RPKM) of each bin is the same. Error bars show standard deviations of the overall read-through rate estimated by bootstrapping genes in each bin. Error bars are too small to be visible for some bins. A total of 3,688 yeast genes and 9,519 fruit fly genes are included in the analysis. Spearman's rank correlation (*ρ*) and associated *P*-value are shown.

To differentiate between the error hypothesis and the adaptive hypothesis, we first examined yeast and fruit fly genes reported by Dunn et al. to undergo stop-codon read-through based on ribosome profiling [9], because different studies used different protocols such that the read-through rates and expression levels estimated in different studies are not directly comparable. We measured the rate of stop-codon read-through in a gene by the number of ribosome-profiling reads per kilobase per million mapped reads (RPKM) in the segment between the canonical stop codon and the following in-frame stop codon in 3’ UTR, relative to that in the coding region. We quantified the expression level of a gene using RPKM in the coding region on the basis of mRNA sequencing. Indeed, the rate of stop-codon read-through is negatively correlated with the gene expression level in both the yeast (Spearman’s *ρ* = -0.49, *P* = 0.01; **Fig 1A**) and fruit fly (*ρ* = -0.45, *P* < 10^−14^; **Fig 1B**). The yeast result confirms that from a recent, independent ribosome-profiling experiment [23]. Note that because the rate of read-through is computed using ribosome profiling data while the gene expression level is computed using mRNA sequencing data, the above correlation is not an artifact of statistical non-independence that has been found to affect a number of gene expression analyses [24]. Nevertheless, because the detectability of stop-codon read-through increases with the mRNA concentration, both low and high rates of read-through are detectable in highly expressed genes while only high rates of read-through may be detected in lowly expressed genes. Thus, a negative correlation between the read-through rate and gene expression level could have resulted simply from this potential detection bias. To rectify this problem, we considered all genes instead of only those reported to undergo stop-codon read-through. Genes were ranked by the expression level and then divided into 10 bins such that each bin contained the same total expression level measured by RPKM from mRNA sequencing. We then computed the overall stop-codon read-through rate of each bin by considering all genes in the bin together as a “supergene” (instead of averaging the read-through rates of individual genes in the bin). The uniformity of the total expression level among bins eliminates the detection bias aforementioned. Yet, we still found that the read-through rate of a bin decreases as the average gene expression level of the bin rises in both the yeast (*ρ* = -0.78, *P* = 0.01; **Fig 1C**) and fruit fly (*ρ* = -1, *P* < 10^−300^, **Fig 1D**). These observations support the error hypothesis of stop-codon read-through.

### Read-through motifs are avoided in highly expressed genes

Manipulative experiments showed that the rate of stop-codon read-through depends on the specific stop codon and its flanking sequence [4, 12, 25–27]. Here, we investigate the frequencies of motifs TGACA and TGACT (stop codons underlined), which are highly susceptible to stop-codon read-through [12, 26]. The error hypothesis predicts that read-through motifs should be selected against, especially in highly expressed genes, due to the larger harm of stop-codon read-through in more highly expressed genes. By contrast, the adaptive hypothesis does not predict *a priori* an underrepresentation of read-through motifs in highly expressed genes.

**Fig 2:**
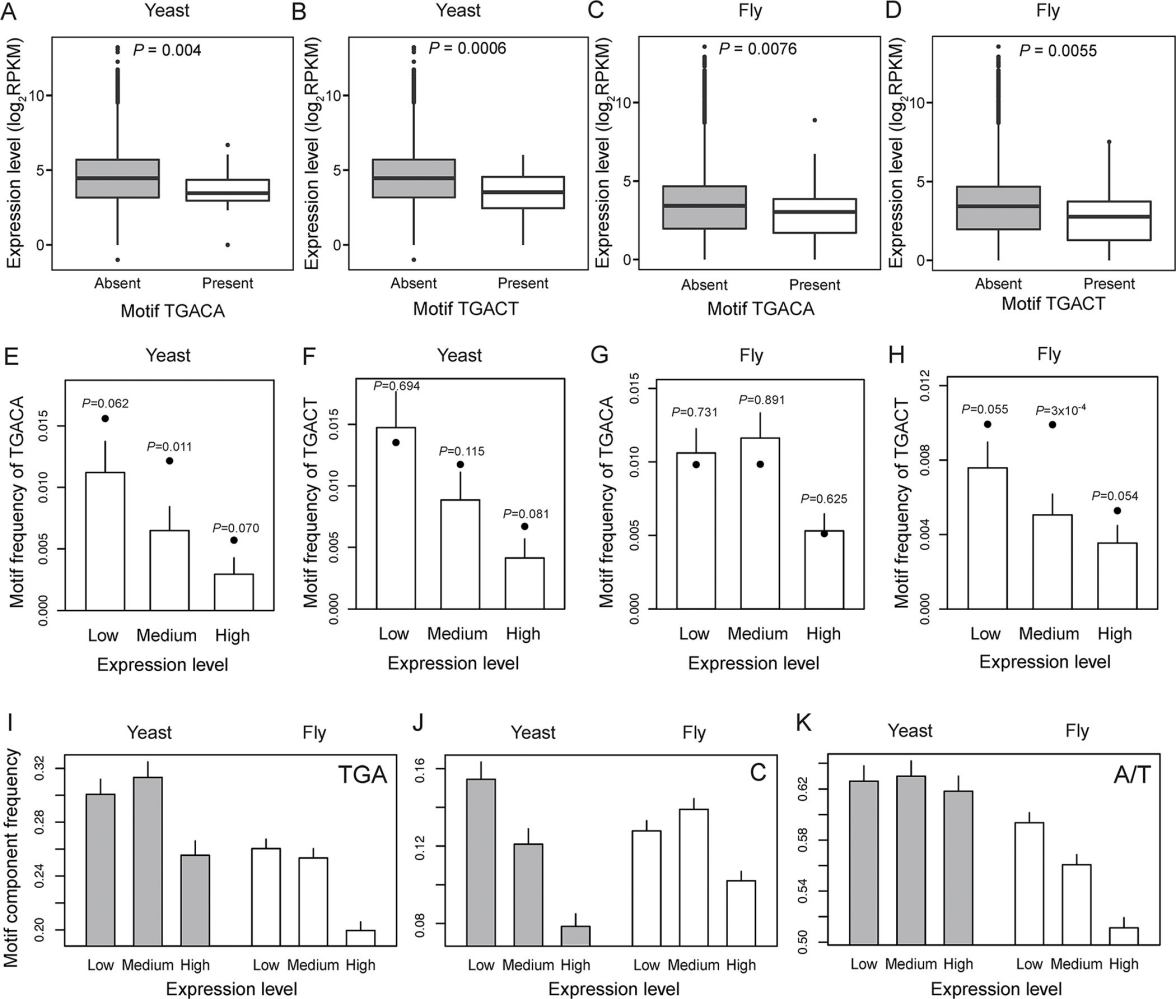
Read-through motifs are avoided in highly expressed genes. “TGACA” and “TGACT” (stop codon underlined) are two previously identified sequence motifs conducive to stop-codon read-through. A total of 6,572 yeast genes and 11,895 fruit fly genes are considered. (**A**-**D**) Yeast (**A**-**B**) and fruit fly (**C**-**D**) genes with and without the read-through motifs show significantly different expression levels. The distribution of gene expression level is shown in a box plot, where the lower and upper edges of a box represent the first (qu_1_) and third quartiles (qu_3_), respectively, the horizontal line inside the box indicates the median (md), the whiskers extend to the most extreme values inside inner fences, md±1.5(qu_3_-qu_1_), and the circles represent values outside the inner fences (outliers). *P*-values are based on Mann-Whitney U tests. (**E**-**H**) Observed and expected motif frequencies in yeast (**E**-**F**) and fruit fly (**G**-**H**) genes of low, medium, and high expressions. Genes are ranked by expression levels and then divided into three bins of equal numbers of genes. Each bar shows the observed motif frequency, with the error bar representing the standard deviation based on 1,000 bootstrap samples of genes within the bin. Each dot shows the motif frequency expected from the observed frequencies of the three components (stop codon and each of the following two nucleotides) of the motif in the bin. We shuffled motif components among genes in the same bin 10,000 times, and the *P*-value above each dot shows the probability that the number of motifs observed upon a shuffle is equal to or smaller than the number in the actual genes. (**I**-**K**) Frequencies of the stop codon TGA (**I**), C following the stop codon (**J**), and A/T at the next position (**K**) in yeast (grey bars) and fruit fly (white bars) genes of low, medium, and high expressions. Error bars show standard deviations based on 1,000 bootstrap samples of genes within the bin. Note that the Y-axis does not start from 0 in (I)-(K).

We found the expression levels significantly lower for yeast genes containing TGACA (*P* = 0.004, Mann-Whitney U test; **Fig 2A**) or TGACT (*P* = 0.0006; **Fig 2B**) than those without such motifs. Similar results were obtained in the fruit fly (*P* = 0.008 and 0.006, respectively; **Fig 2C** and **2D**). Thus, the read-through motifs are underrepresented in highly expressed genes when compared with lowly expressed genes. Consistently, when genes are divided into three equal-size bins with low, medium, and high expressions, the frequencies of the read-through motifs generally decrease as the expression increases (bars in **Fig 2E–2H**).

Two mechanisms might account for the underrepresentation of read-through motifs in highly expressed genes. First, each component (i.e., stop codon TGA, C after the stop codon, and A/T at the next position) of the read-through motifs may be underrepresented. Second, the combinatory use of the three components may be underrepresented relative to the expectation from the frequencies of the three components. There is clear evidence for the first mechanism. For instance, in both the yeast and fruit fly, the 20% most highly expressed genes use the stop codon TGA significantly less often than the rest of the genes (*P* < 0.005, Fisher's exact test). A systematic analysis shows that the frequency of each of the three components is lower in the high-expression bin than in the low-expression bin in the yeast and fruit fly, and all these differences are statistically significant except for the second position after stop codon in the yeast (**Fig 2I–2K**). Consequently, the expected frequency of a motif, computed from the product of the frequencies of the three components of the motif in a bin, is lower for the high-expression bin than the low-expression bin for each motif in each species (dots in **Fig 2E**–**2H**). To probe the second mechanism, we compared the observed frequency of a read-through motif with the above computed expected value. There is some evidence for a significant deficiency of the observed frequency relative to the expected frequency for motif TGACA in the yeast (**Fig 2E**) and motif TGACT in the fruit fly (**Fig 2H**), demonstrating the presence of the second mechanism. The absence of read-through motifs with higher-than-expected frequencies is inconsistent with the contention that stop-codon read-through is selectively favored. Because the above findings are based on genome sequences, they complement the results from ribosome-profiling data that are limited by the condition or cell type used in the experiments.

### Read-through regions do not show increased sequence conservation

If stop-codon read-through mostly results from molecular errors, the post-stop-codon translated region should not be evolutionarily more conserved than comparable regions that are untranslated. By contrast, the adaptive hypothesis predicts that the translated region should be more conserved. Below, we examine these contrasting predictions by interspecific comparisons between *S*. *cerevisiae* and *S*. *paradoxus* and between *D*. *melanogaster* and *D*. *simulans*. We restricted our comparison to closely related species because interspecific conservation of stop-codon read-through is limited [8]. Specifically, let region 1 be the transcript segment between the canonical (i.e., first) stop codon and the next (i.e., second) in-frame stop codon, and let region 2 be the transcript segment between the second and third in-frame stop codons. Region 1 is translated in genes subject to stop-codon read-through but not in other genes, whereas region 2 should be untranslated except in the rare case of double read-through. Because genes with and without stop-codon read-through may differ in aspects other than stop-codon read-through, respectively comparing their sequence conservations for region 1 and region 2 allows testing signals of sequence conservation specifically associated with the read-through. Sequence conservation is measured by percent nucleotide sequence identity at aligned non-gapped sites. In the following analyses, we considered yeast and fly genes previously reported on the basis of ribosome-profiling to undergo read-through [8, 9]. We combined the read-through genes of yeast from two studies [8, 9] to increase the statistical power.

**Fig 3:**
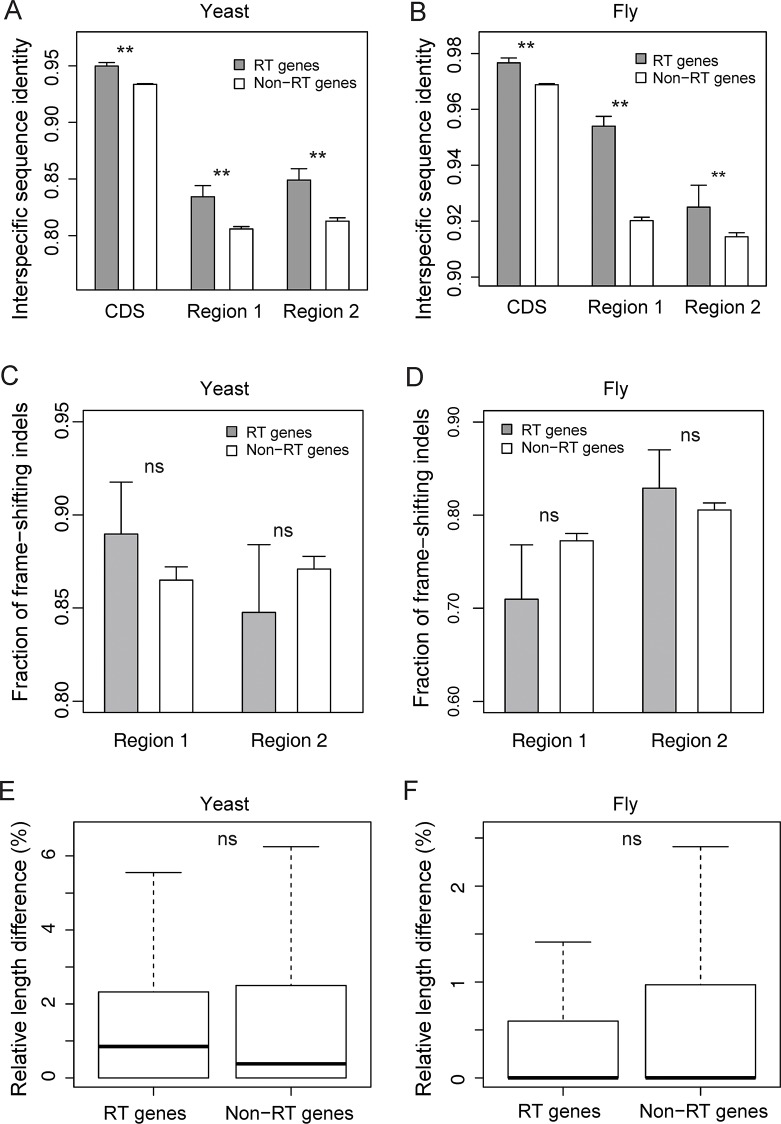
Conservations of various regions in read-through (RT) and non-read-through (non-RT) genes. The read-through genes used are from [8, 9], whereas all other genes are treated as non-read-through genes. A total of 147 read-through and 4,225 non-read-through yeast genes and 233 read-through and 8,367 non-read-through fly genes are considered. Region 1 refers to the transcript segment between the canonical stop codon and the following in-frame stop codon, whereas region 2 refers to the transcript segment between the first and second in-frame stop codon after the canonical stop codon. CDS, coding sequence. (**A**-**B**) Sequence conservation of coding region, region 1, and region 2 between *S*. *cerevisiae* and *S*. *paradoxus* (**A**) or between *D*. *melanogaster* and *D*. *simulans* (**B**) for read-through and non-read-through genes. (**C**-**D**) Fractions of frame-shifting indels in regions 1 and 2 of read-through and non-read-through genes, based on a comparison between the two yeasts (**C**) or two fruit flies (**D**). In (A)-(D), error bars show the standard deviation of the mean determined by bootstrapping genes. Each one-tailed *P*-value is based on respectively bootstrapping genes of the two groups being compared, and "ns" means not significant. (**E**-**F**) Relative length differences in regions 1 and 2 of read-through genes and non-read-through genes between the two yeasts (**E**) or two fruit flies (**F**). In each boxplot, the lower and upper edges of a box represent the first (qu_1_) and third quartiles (qu_3_), respectively, the horizontal line inside the box indicates the median (md), and the whiskers extend to the most extreme values inside inner fences, md±1.5(qu_3_-qu_1_). One-tailed Mann-Whitney U test result is presented, where "ns" means not significant. Note that the Y-axis does not start from 0 in some panels.

Between the two yeasts, region 1 is more conserved in genes reported to undergo read-through than other genes, but region 2 exhibits a similar trend (**Fig 3A**). Thus, the higher sequence conservation of read-through genes than non-read-through genes in region 1 may not be related to the read-through. Similar results were obtained in the two fruit flies (**Fig 3B**). In the fruit flies, because the excess in sequence conservation of read-through genes looks greater for region 1 than region 2, we further examined the conservations of three codon positions respectively. If the sequence conservation in these regions is due to any protein-level function, we expect first two codon positions to be more conserved than third codon positions because mutations are less likely to be neutral at first two codon positions than at third codon positions [28]. However, we found that first two codon positions are no more conserved than third codon positions in region 1, regardless of whether the genes are subject to stop-codon read-through (*P* = 0.42) or not (*P* = 0.088). The same pattern applies to region 2 (*P* = 0.48 and 0.11, respectively). The above *P*-values were determined by bootstrapping relevant genes 1000 times and computing the fraction of bootstrap samples where first two codon positions are less conserved than or equally conserved as third codon positions. It is worth noting that, in both yeasts and fruit flies, read-through genes are more conserved than non-read-through genes in all three regions examined (coding region, region 1, and region 2) (**Fig 3A** and **3B**). This is probably because read-through genes tend to be relatively highly expressed as a result of the detection bias aforementioned and because sequence conservation tends to be greater in more highly expressed genes at least for coding regions [29] and it may also be true for 3’ UTRs due to evolutionary constraints of regulatory sequences.

To compare read-through genes with non-read-through genes of similar expression levels, we ranked all genes based on their expression levels. For each read-through gene, we picked two non-read-through genes as controls, one immediately behind and one immediately ahead of the read-through gene in the ranking, and computed the mean between-species sequence conservation of the two controls. We found no significant difference in the sequence conservation of region 1 between the yeast read-through genes and non-read-through genes of similar expression levels (*P* = 0.93, paired *t*-test). In the fruit fly, read-through genes are significantly more conserved than non-read-through genes of similar expression levels (*P* = 0.024), but the significance disappears (*P* = 0.053) upon the exclusion of only three genes (FBgn0036994, FBgn0043010, and FBgn0016926), suggesting that, for the vast majority of read-through genes, there is no enhanced conservation of region 1.

### Frame-shifting indels are not underrepresented in read-through regions

After investigating the aligned non-gapped sites, we turned to insertions/deletions (indels) in regions 1 and 2. If post-stop-codon translation is functional, frame-shifting indels (i.e., not of multiples of 3 nucleotides) should be deprived in translated regions, while no such trend is expected under the error hypothesis. We found no significant differences in the proportion of frame-shifting indels between genes with and without read-through in either region 1 or 2 of either species pair examined (**Fig 3E** and **3F**). These results are consistent with the error hypothesis, but are inconsistent with the adaptive hypothesis that predicts an underrepresentation of frame-shifting indels specifically in region 1 of the read-through genes.

### Read-through regions show no increased length conservation

The adaptive hypothesis further predicts conservation of the length of region 1 in genes undergoing read-through, while no such prediction is made by the error hypothesis. We first computed the absolute interspecific length differences of region 1 for genes with and without read-through. Between the two yeasts, the average length differences are 2.67 and 2.30 nucleotides (nt) for read-through and non-read-through genes, respectively (*P* = 0.014, two-tailed Mann-Whitney *U* test). This result is opposite to the prediction of the adaptive hypothesis. Between the two fruit flies, the average length differences are 1.56 and 2.10 nt for read-through and non-read-through genes, respectively (*P* = 0.14). While in the direction predicted by the adaptive hypothesis, this disparity is not statistically significant.

We noticed that region 1 is on average longer in read-through genes (65.42 nt in *S*. *cerevisiae* and 62.31 nt in *D*. *melanogaster*) than non-read-through genes (46.69 nt in *S*. *cerevisiae* and 52.37 nt in *D*. *melanogaster*). This observation is likely due to detection bias, because read-through is more detectable by ribosome profiling when region 1 is longer. Hence, it may not serve as evidence for the functionality of read-through regions. To correct for this bias in the comparison of interspecific length differences, we computed the relative length difference of region 1 between species for genes with and without read-through, respectively. The relative length difference is the absolute value of (*L*_A_−*L*_B_)/(*L*_A_ + *L*_B_), where *L*_A_ and *L*_B_ are the lengths of the orthologous region 1 in the two species compared. In neither the yeasts (*P* = 0.15, Mann-Whitney *U* test; **Fig 3E**) nor fruit flies (*P* = 0.15; **Fig 3F**) was the relative length difference significantly different between genes with and without read-through. Thus, regardless of whether the absolute or relative length difference between species is considered, read-through genes show no increased region 1 length conservation than non-read-through genes.

### The harm of read-through is alleviated by reduced hydrophobicity of the extended peptide

**Fig 4:**
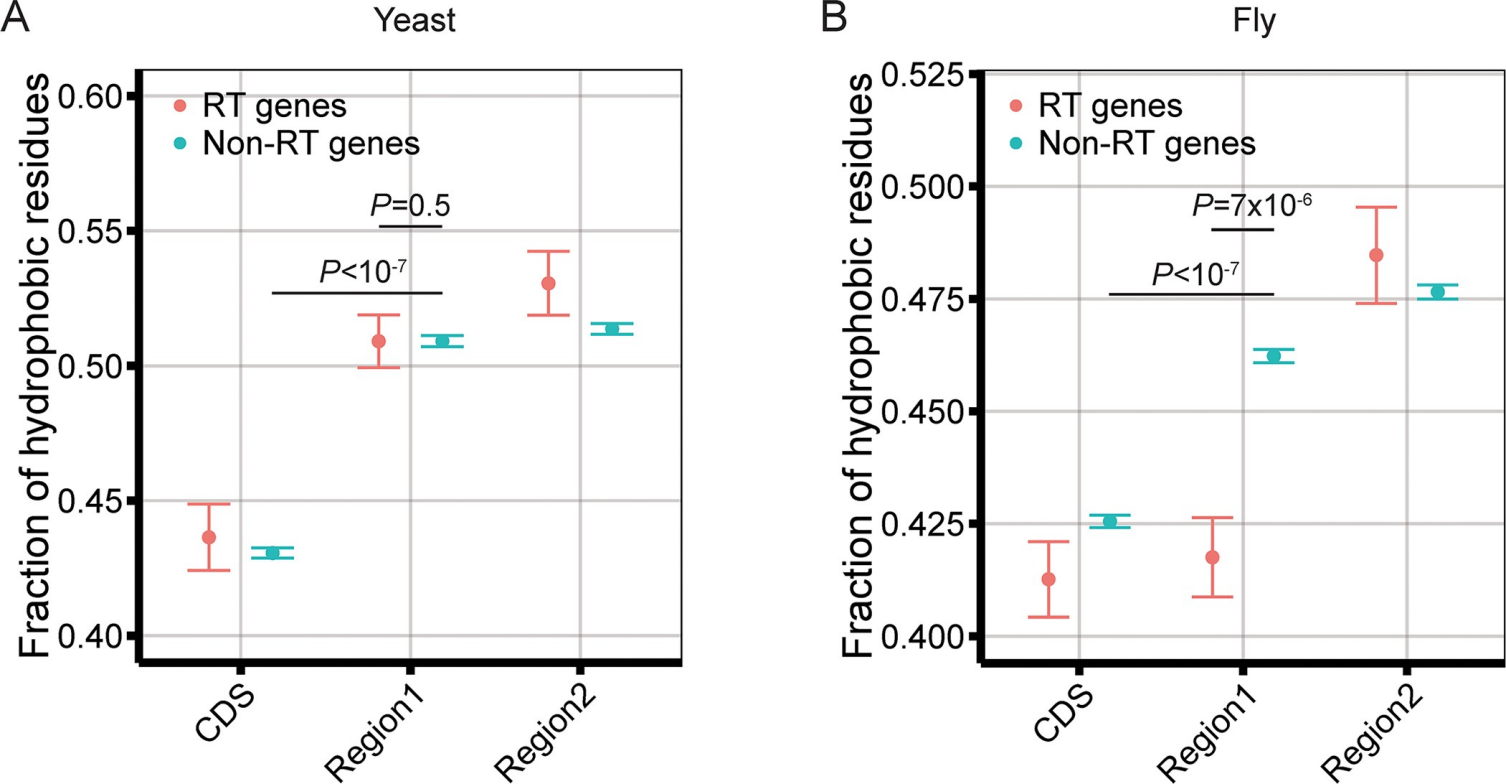
Read-through (RT) regions have reduced amino acid hydrophobicity compared with non-read-through (non-RT) regions. Presented are the fractions of hydrophobic amino acids in the peptides conceptually translated from the last 16 codons of the coding region (CDS), region 1, and region 2 in 172 read-through genes and 6,399 non-read-through genes of the yeast (**A**) or 294 read-through genes and 11,389 non-read-through genes in the fruit fly (**B**). Error bars show standard errors determined by bootstrapping genes. One-tailed *P*-value is based on respectively bootstrapping genes of the two groups being compared.

Together, the above analyses strongly support the hypothesis that stop-codon read-through mostly results from molecular errors. Recent experiments in nematode and human cells showed that artificially-made fusion proteins corresponding to the coding region and region 1 combined tend to be unstable and degraded when compared with the proteins corresponding to the coding region only [30], a clear indication that read-through would be deleterious. The experiments, however, did not focus on genes with natural stop-codon read-through. Thus, the results suggest that stop-codon read-through in these genes, which probably have low read-through rates naturally, is deleterious. The study also found that the deleterious effect of translation of region 1 increases with the hydrophobicity of the peptide corresponding to region 1 [30], presumably because the hydrophobic residues translated from region 1 interfere with protein folding. Given these observations, we predict that natural selection minimizing the harm of stop-codon read-through may result in a lower hydrophobicity of the peptide corresponding to region 1 of read-through genes than that corresponding to non-read-through genes, either because natural selection drives the decrease in hydrophobicity of the extended peptide or because natural selection disfavors the read-through of genes where the extended peptide would be highly hydrophobic. To this end, we first compared the hydrophobicity of the coding region and region 1 of non-read-through genes. We found the former to be significantly lower than the latter in both the yeast (**Fig 4A**) and fruit fly (**Fig 4B**), confirming that functional proteins are required to have a lower hydrophobicity than the random expectation, which is represented by region 1 of non-read-through genes. In support of our prediction, the hydrophobicity of region 1 is indeed lower for read-through genes than non-read-through genes, although the trend is significant in the fruit fly (**Fig 4B**) but not in the yeast (**Fig 4A**). This difference between the fruit fly and yeast may be related to the fact that the read-through rate is overall much lower in the yeast than in the fruit fly (**Fig 1**). The deleterious effects of molecular errors could be alleviated by global solutions such as a reduction in the overall read-through rate via ribosomal improvements or by local solutions such as a reduction in the hydrophobicity of the read-through peptide of a given gene [31]. It is possible that more global solutions have evolved in yeast while more local solutions have appeared in the fruit fly. Theory predicts that both solutions are possible in the yeast and fruit fly due to their huge effective population sizes [31]. At any rate, our finding of a lowered hydrophobicity of region 1 of read-through genes reveals natural selection minimizing the harm of stop-codon read-through and hence further supports the error hypothesis. As a control, we also examined region 2, which is translated in neither read-through nor non-read-through genes. In the yeast, regions 1 and 2 behave similarly in hydrophobicity for both read-through and non-read-through genes, consistent with the above interpretation. In the fly, region 2 of both read-through and non-read-through genes has a high hydrophobicity, similar to that of region 1 of non-read-through genes, as expected.

## Discussion

In this work, we used ribosome-profiling data from the unicellular model fungus *S*. *cerevisiae* and multicellular model animal *D*. *melanogaster* and genome sequences from these and other species to test the hypothesis that stop-codon read-through results largely from molecular errors and is generally nonadaptive. In both the yeast and fruit fly, we observed that (i) the read-through rate decreases with the level of gene expression, (ii) sequence motifs conducive to read-through are underrepresented among highly expressed genes, and (iii) read-through regions do not exhibit increased sequence conservation, avoidance of frame-shifting indels, or resistance to length changes. Furthermore, it was previously reported that most read-through events are not conserved between species [8]. Together, these observations strongly support the error hypothesis and reject the assertion that most read-through events are functional and adaptive.

To estimate the fraction of read-through events that are deleterious, we followed two recent studies [21, 32]. Stop-codon read-through is minimally selected against in lowly expressed genes because the waste of energy and production of toxic products are minimal. Hence, the read-through rate in lowly expressed genes may be considered the intrinsic read-through rate without selective minimization. Following the same logic, stop-codon read-through in highly expressed genes is comparatively costly so has been selectively minimized. Hence, the read-through rate observed in highly expressed genes reflects the rate of non-deleterious read-through upon the selective removal of deleterious read-through. So, the amount of read-through in highly expressed genes that has been removed by natural selection can be estimated from the difference in read-through rate between lowly and highly expressed genes. The read-through rate is 0.0398 in the leftmost bin and 0.0111 in the rightmost bin in Fig 1C. Hence, we estimate that (0.0398–0.0111)/0.0398 = 72.1% of read-through has been removed by natural selection in highly expressed yeast genes. The corresponding value is (0.04069–0.01318)/0.04069 = 67.6% in highly expressed fruit fly genes. That a slightly larger fraction of read-through has been selectively removed in the yeast than the fruit fly is expected, because the effective population size is larger in the yeast, so the efficacy of natural selection is greater in the yeast than in the fruit fly [33, 34]. The above percentages are conservative estimates of the fraction of deleterious read-through events, because slightly deleterious read-through may not have been fully removed by selection in highly expressed genes and because some strongly deleterious read-through may have been removed by selection even in lowly expressed genes. If the probability that stop-codon read-through is harmful is the same in lowly and highly expressed genes in spite of a difference in the magnitude of the harm, we would conclude that at least 72% and 68% of read-through events are deleterious in yeast and fruit fly, respectively.

In light of our finding, it is worth reexamining evidence previously thought to support the adaptive hypothesis of stop-codon read-through. First, the read-through rate is known to increase under some stresses, which could be advantageous when new proteins able to cope with the stresses are needed [14, 15]. One mechanism of the stress-induced read-through in yeast is the conversion of the release factor Sup35 from its normal folding to an aggregated amyloid conformation known as the prion state, which induces more Sup35 molecules to convert to the prion state, causing rampant stop-codon read-through [35, 36]. Although such an elevation in the read-through rate may be an active response to stress, it could also be a passive, deleterious consequence because cells are not in their optimal physiological state under stress. Interestingly, comparing the growth rates between two yeast strains of the same genetic background, one with a higher read-through rate than the other, across multiple stressful environments showed that high read-through is advantageous in some environments but disadvantageous in some other environments [35]. Further, a simulation study showed that selection against the Sup35 prion appearance is substantial [37]. Some researchers contended that, although stop-codon read-through may not be particularly beneficial in the current environment, it may eventually lead to higher fitness in the long run because it reveals cryptic protein-coding sequences in the UTR under stresses [31]. However, experimental evolution showed that the rate of yeast adaptation to new environments is not necessarily higher with the Sup35 prion than without the prion [38]. Furthermore, even if stop-codon read-through improves evolvability, read-through could still be errors, analogous to genetic mutations, which are errors that could increase evolvability. As mentioned, another line of evidence supporting the adaptive hypothesis was the observation that region 1 is significantly more conserved than region 2 among fruit fly read-through genes [9]. Our analysis showed that while this is true in fruit flies (**Fig 3B**), it is not true in yeasts (**Fig 3A**). Furthermore, in fruit flies, region 1 is more conserved than region 2 even in non-read-through genes (**Fig 3B**), suggesting the possibility that the excess conservation of region 1 is not related to read-through. Indeed, in both yeasts and flies, we found no excess in region 1 sequence conservation for all or the vast majority of read-through genes when compared with non-read-through genes of similar expression levels. In addition, the analyses of frame-shifting indels and sequence length evolution support that, in both yeasts and fruit flies, region 1 does not have stronger selective constraints in read-through genes than non-read-through genes. In this context, it is worth mentioning that some authors use sequence conservation of region 1 to identify potential read-through genes under the premise that the conservation would imply functional read-through [39, 40]. Our finding that sequence conservation of region 1 may not be related to read-through cautions against this practice. It is notable that only 15% of fruit fly read-through events predicted by sequence conservation of region 1 were confirmed in ribosome profiling (although this could be due to limited sampling of tissues or developmental stages), while only 12% of read-through events observed in ribosome profiling were predicted from sequence conservation [9]. These small overlaps are consistent with our conclusion that most read-through regions are not conserved and most conserved region 1 sequences are not subject to read-through.

Following previous transcriptome-wide studies of stop-codon read-through [8, 9], we used ribosome profiling to identify such events. Although not every ribosome footprint indicates translation, a reasonably high fraction of read-through events identified by ribosome profiling are verifiable at the protein level [9]. Furthermore, even if ribosome profiling produces some false read-through signals, such errors cannot explain our observation of a negative correlation between gene expression level and read-through rate (**Fig 1**), unless transcripts of lowly expressed genes have more ribosome protections than those of highly expressed genes. To the best of our knowledge, no such bias has been reported or is expected.

That most read-through events are nonadaptive does not preclude the possibility that a small proportion of such events have been co-opted in evolution for certain functions. It will be of interest to identify such functional cases from the sea of largely functionless read-through events. We suggest that such functional cases are likely conserved among multiple species, have high read-through rates, and show multiple signals of functional constraints in region 1 such as reduced sequence variation among species and avoidance of frame-shifting indels. Candidates for adaptive read-through can be experimentally verified by measuring the functional and/or fitness effect of altering the read-through rate, for example, by modifying the read-through motif. While laborious, this approach can provide definitive evidence for adaptive read-through.

Our results support the hypothesis that most stop-codon read-through events are one type of translational error, which also includes the incorporation of erroneous amino acids in protein synthesis (i.e., mistranslation). It has been estimated that the mistranslation rate ranges from 10^−5^ to 10^−2^ per codon, depending on the type of error [41, 42]. We found the stop-codon read-through rate between 10^−4^ and 10^−2^ in the yeast and fruit fly, depending on the gene expression level. Thus, the read-through rate is generally consistent with the mistranslation rate, and both of them are higher than the rate of transcriptional error [43]. Several recent studies showed that a number of cellular processes that are widely thought to be beneficial for generating transcriptomic and proteomic diversities, such as alternative transcriptional initiation, alternative splicing, alternative polyadenylation, and various RNA modifications, result largely from molecular errors and are generally nonadaptive [17–21, 32]. That random errors are not uncommon even in the seemingly exquisitely regulated and optimized processes of RNA and protein synthesis reminds us of the inherent stochasticity and imprecision of the cellular life. It also cautions against assuming adaptive values of any phenomenon without critical evaluation, even if the phenomenon is common at the genomic scale.

## Materials and methods

### Data source

The genome and gene sequences of *Drosophila* and *Saccharomyces* species were downloaded from the publicly available SGD [44], FlyBase [45], and Ensembl [46] databases. The ribosome-profiling data and read-through rates of *D*. *melanogaster* and *S*. *cerevisiae* genes were from Dunn et al. [9]. The list of 350 read-through genes in *D*. *melanogaster* was from the same study [9]. When evaluating the general properties of read-through genes, a list of 172 read-through genes in *S*. *cerevisiae* was used, which is the union of the read-through genes from two previous studies [8, 9].

Gene expression data of *D*. *melanogaster* were downloaded from FlyBase (FB2016_04) [45]. The mean expression level of a gene across multiple growth stages was used as a proxy for its overall expression level [47]. When calculating the log_2_(RPKM) for individual genes in Fig 2C and 2D, we added 1 to the RPKM of all genes to avoid undefined log_2_(RPKM) when RPKM = 0. Gene expression levels in *S*. *cerevisiae* were from the study by Nagalakshmi *et al*. [48].

### Read-through rates of groups of genes with different expression levels

Read-through cannot be detected in lowly expressed genes unless the read-through rate is high, creating an artifactual negative correlation between gene expression level and read-through rate. We designed the following method to rectify this problem. We first ranked all genes by their expression levels measured by the RPKM of the coding region from mRNA sequencing data mentioned in the above section. Based on the ranking, we then grouped these genes into ten bins, requiring the total RPKM for each bin to be equal. We computed an overall read-through rate of each bin by the sum of RPKM of region 1 divided by the sum of RPKM of coding regions from the ribosome-profiling data. The standard error of the read-through rate was calculated by bootstrapping genes in each bin 1,000 times. The yeast and fly RPKM data from both ribo-seq and mRNA-seq were from Dunn et al. [9]. All genes with measured RPKM in CDS and region 1 (including 0 RPKM) were included in this analysis.

### Frequencies of read-through motifs

From each species considered, we respectively estimated the numbers of two read-through motifs, TGACA and TGACT, from all genes in the genome. To understand the underlying mechanisms for the underrepresentation of the motifs in highly expressed genes, we separately shuffled the three motif components (the stop codon and the two nucleotides after the stop codon) among all genes in the same expression bin and respectively counted the numbers of genes with TGACA and TGACT motifs upon the shuffling. This process was repeated 10,000 times to test if the actual number of motifs differs significantly from that expected under a random combinatory use of the three motif components.

### Sequence conservation of one-to-one orthologous genes

We focused on one-to-one orthologs of *S*. *cerevisiae* and its sister species *S*. *paradoxus*, and those of *D*. *melanogaster* and its close relative *D*. *simulans*. For genes with multiple alternative transcripts, the longest transcripts were analyzed. The sequence alignment included regions from 50 nucleotides upstream of the stop codon to 300 nucleotides downstream of the stop codon. To ensure the quality of subsequent analyses, we retained only those alignments for which the stop codon was aligned correctly and the last 36 nucleotides in the alignment had at least 88% sequence identity. We define region 1 by the segment between the canonical (i.e., first) stop codon and the next (i.e., second) in-frame stop codon in the 3’ UTR, and region 2 by the segment between the second and third stop codons in the 3’ UTR based on the sequences in *S*. *cerevisiae* or *D*. *melanogaster*. For region 1 or 2 that extends over the 300-nucleotide length limit, we considered only up to the 300-nucleotide region.

### Evaluating hydrophobicity

We calculated the fraction of hydrophobic sites for the last 16 amino acids of each protein, region 1, and region 2 for 6,517 and 11,683 genes in *S*. *cerevisiae* and *D*. *melanogaster*, respectively. The following amino acids were considered hydrophobic: G, A, V, I, L, M, F, Y, and W.
